# Polyclonal antibody cocktails generated using DNA vaccine technology protect in murine models of orthopoxvirus disease

**DOI:** 10.1186/1743-422X-8-441

**Published:** 2011-09-20

**Authors:** Joseph W Golden, Marina Zaitseva, Senta Kapnick, Robert W Fisher, Malgorzata G Mikolajczyk, John Ballantyne, Hana Golding, Jay W Hooper

**Affiliations:** 1 Virology Division, United States Army Medical Research Institute of Infectious Diseases, Fort Detrick, MD 21702, USA; 2 Division of Viral Products, Center for Biologics Evaluation and Research, Food and Drug Administration, Bethesda, MD 20892, USA; 3 Division of Hematology, Center for Biologics Evaluation and Research, Food and Drug Administration, Bethesda, MD 20892, USA; 4 Aldevron, LLC, Fargo, ND, 5810, USA

**Keywords:** Smallpox, vaccinia immunoglobulin, monoclonal antibody, passive protection, DNA vaccine, polyclonal antibody, bioluminescence

## Abstract

**Background:**

Previously we demonstrated that DNA vaccination of nonhuman primates (NHP) with a small subset of vaccinia virus (VACV) immunogens (L1, A27, A33, B5) protects against lethal monkeypox virus challenge. The L1 and A27 components of this vaccine target the mature virion (MV) whereas A33 and B5 target the enveloped virion (EV).

**Results:**

Here, we demonstrated that the antibodies produced in vaccinated NHPs were sufficient to confer protection in a murine model of lethal *Orthopoxvirus *infection. We further explored the concept of using DNA vaccine technology to produce immunogen-specific polyclonal antibodies that could then be combined into cocktails as potential immunoprophylactic/therapeutics. Specifically, we used DNA vaccines delivered by muscle electroporation to produce polyclonal antibodies against the L1, A27, A33, and B5 in New Zealand white rabbits. The polyclonal antibodies neutralized both MV and EV in cell culture. The ability of antibody cocktails consisting of anti-MV, anti-EV, or a combination of anti-MV/EV to protect BALB/c mice was evaluated as was the efficacy of the anti-MV/EV mixture in a mouse model of progressive vaccinia. In addition to evaluating weight loss and lethality, bioimaging technology was used to characterize the spread of the VACV infections in mice. We found that the anti-EV cocktail, but not the anti-MV cocktail, limited virus spread and lethality.

**Conclusions:**

A combination of anti-MV/EV antibodies was significantly more protective than anti-EV antibodies alone. These data suggest that DNA vaccine technology could be used to produce a polyclonal antibody cocktail as a possible product to replace vaccinia immune globulin.

## Background

Naturally occurring smallpox has been eradicated. However, the possibility that smallpox, caused by variola virus (VARV), or a genetically engineered *Orthopoxvirus*, might be reintroduced through a nefarious act remains a low-probability, but high-impact threat. Additionally, monkeypox virus (MPXV) is an emerging virus that causes endemic disease in central Africa and cowpox has caused sporadic serious cases of disease in Europe. These zoonotic viruses have the potential to spread and cause morbidity and mortality in animals and humans [1-4]. Examples of such unexpected long-range spread of these diseases include the monkeypox outbreak in midwestern United States [5] and the recent cowpox outbreaks in Germany [6]. Currently licensed medical countermeasures to prevent *Orthopoxvirus *disease include a live-virus vaccine [7], and vaccinia immune globulin intravenous (VIGIV) to treat adverse events associated with that vaccine [8].

The licensed smallpox vaccine (ACAM2000) is comprised of live-vaccinia virus (VACV) delivered to the skin using a bifurcated needle [7,9]. The health risks associated with live virus vaccination (e.g., ACAM2000) [10,11] necessitate that supplies of VIGIV be available in sufficient quantities to treat certain adverse events associated with the vaccine including eczema vaccinatum, progressive vaccinia, severe generalized vaccinia, VACV infections in individuals who have skin conditions, and other aberrant VACV infections [12]. VIGIV is a US-licensed drug manufactured by the fractionation of hyperimmune plasma derived from persons vaccinated with the live-VACV vaccine [13]. While vaccinia immune globulins have been used in various forms for decades [14-17], efficacy has not been demonstrated in placebo-controlled clinical trials due both to the rare nature of vaccinia-related adverse events and ethical concerns regarding withholding of potentially effective treatments [13]. As is the case with nearly all polyclonal products, the relative protective contribution of the individual antibodies that compose VIGIV are not well understood. Because the hyperimmune plasma is obtained from persons vaccinated with ACAM2000, it contains not only protective antibodies, but also VACV-specific antibodies that do not contribute to protective immunity. It may be possible to replace this immunotherapeutic with a more defined product comprised of a cocktail of polyclonal or monoclonal antibodies targeting key protective epitopes in VACV.

Only a small subset of the ~200 open reading frames in the *Orthopoxvirus *genome encode proteins that have been implicated in protective immunity. Most of these proteins are found on the surfaces of the two infectious forms of orthopoxviruses: the mature virion (MV) and the extracellular enveloped virion (EV). Targets include the MV proteins encoded by the L1R, A27L, D8L, H3L open reading frames; and the EV proteins encoded by A33R and B5R [18-39]. Studies involving active vaccination with protein- or gene-based subunit vaccines, as well as passive transfer studies using monoclonal antibodies, have found that combinations of MV and EV targets afford improved protection over MV or EV alone [24,30,31].

Based on the safety profile of Dryvax, ACAM2000, and other live-vaccinia-based vaccines [7,10,11], a safer (poorly replicating) smallpox vaccine would be ideal, especially for at-risk populations. One such vaccinia strain, modified vaccinia Ankara (MVA) has been the focus of extensive research to determine if it is an acceptable alternative to existing vaccinia strains. MVA and its derivative strains are highly attenuated, and undergo limited replication in primate cells [40]. While the MVA-based vaccines are immunogenic and have a favorable safety profile, higher doses of vaccine and multiple administrations of vaccine are required to achieve adequate titers. Moreover, the duration of immunity (both humoral and cellular) remains a concern with the MVA-based vaccine candidates. An alternative approach for the development of safer yet efficacious vaccines is to avoid the use of live-virus-based vaccines entirely and instead identify specific subunits or epitopes from *Orthopoxvirus *species that confer protection and vaccinate with those subunits. Protein- and gene-based subunit vaccines against orthopoxviruses have been investigated by a number of groups [18-39,41-44]; for review see [45]. Our laboratory has focused on gene-based vaccines involving a combination of two MV and two EV targets. The four-target combination (L1R, A27L, A33R, and B5R) has been termed 4pox for simplicity. This vaccine delivered by various technologies has protected mice, rabbits, and nonhuman primates (NHP) against VACV [28,31,33], rabbitpox virus (Hooper, J.W., et al manuscript forthcoming), and monkeypox virus [32,34] and Golden, J.W., et al manuscript forthcoming). Here, we investigated the protective efficacy of antibodies elicited by the 4pox DNA vaccine. We found that serum from immune NHP or rabbits could provide complete protection from lethality in VACV intranasal murine challenge models, as well as partial protection in a mouse model of progressive vaccinia. Previously, we showed that wholebody bioimaging technology can be used to follow replication of TK^+ ^recombinant WRvFire and IHD-J-Luc vaccinia strains expressing luciferase reporter gene in live mice [46,47]. Using this model system, we explored how anti-MV and anti-EV polyclonal sera limit VACV dissemination in mice. Our findings demonstrate that cocktails comprised of a combination of anti-EV polyclonal antibodies are more effective than anti-MV antibodies in preventing virus replication and dissemination from the nasal cavity to lungs, spleen and liver. However, the most significant protection from weight loss and pox lesion development was achieved by a combination of anti-EV and anti-MV polyclonal antibodies. This proof-of-concept study revealed that DNA vaccine technology can indeed be used to make a cocktail of polyclonal antibodies that can be used as a viable immunotherapeutic to treat *Orthopoxvirus *disease.

## Results

### Sera from NHP vaccinated with the 4pox DNA vaccine protected mice against lethal respiratory challenge with VACV

**Figure 1:**
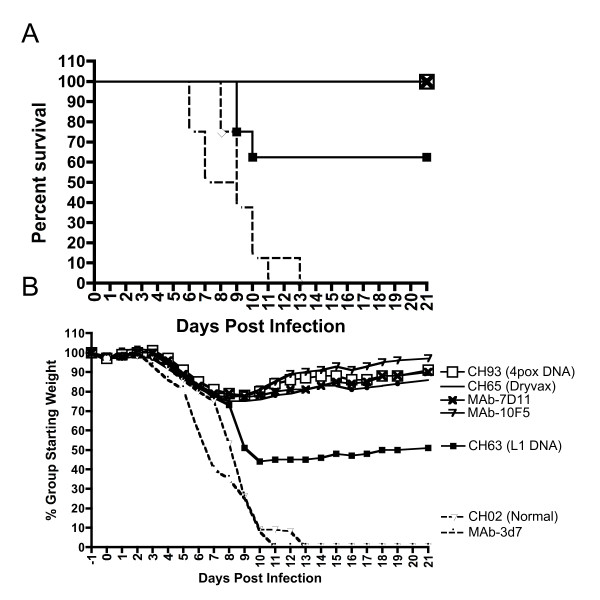
**Sera from vaccinated NHP can protect mice from lethal VACV challenge**. Groups of eight mice were injected subcutaneously with heat-inactivated sera collected from NHP previously vaccinated [34] with Dryvax (NHP ID# CH63 ref. x), 4pox DNA vaccine (NHP ID# CH93), L1R DNA vaccine (NHP ID# CH63), or an irrelevant DNA vaccine (NHP ID# CH02). As positive controls for protection, groups of eight mice were injected with 100 μg of purified anti-L1 murine monoclonal antibodies MAb-7D11 or MAb-10F5. As negative controls, eight mice received 100 μg of an irrelevant murine monoclonal antibody (MAb-3d7). One day after injection with NHP sera or murine MAbs, the mice were challenged with 2 × 10^6 ^PFUPFU of VACV strain IHD-J intransally. Percent survival (**A**) and percent group weight loss (**B**) were plotted. In this experiment, individual mouse weights were not recorded.

Previously, we demonstrated that it was possible to vaccinate NHP with the 4pox (L1, A27, B5, A33) DNA vaccine using various gene-based delivery technologies and protect against lethal MPXV [32,34]and Golden, J.W., et al manuscript in forthcoming). To test whether the humoral responses produced by these vaccines were sufficient to confer protection, we tested sera from NHP vaccinated with either the 4pox DNA vaccine (L1, A33, B5 and A33), L1R-alone or Dryvax, for the capacity to protect mice from lethal VACV infection. Normal monkey sera (NMS) and murine monoclonal antibodies were included as controls. Sera were injected subcutaneously 1 day before challenge with VACV. The 4pox and Dryvax sera protected all mice from lethal disease (Figure 1A). In contrast, sera from NHP vaccinated with the L1 DNA only protected 50% of the mice, and all of the animals receiving NMS sera or a negative control mouse MAb succumbed to disease. Weight loss in mice injected with the 4pox or Dryvax sera was similar to the weight loss in mice receiving 100 μg of L1-specific positive controls MAb-7D11 or MAb-10F5, indicating similar mild disease in these groups (Figure 1B). These data indicated for the first time that sera from a NHP vaccinated with a molecular smallpox vaccine can confer protection in a model of orthopoxvirus disease.

### Vaccination of rabbits with L1R, A27L, B5R and A33L DNA vaccines by muscle electroporation elicits antibody responses against each target immunogen

**Figure 2:**
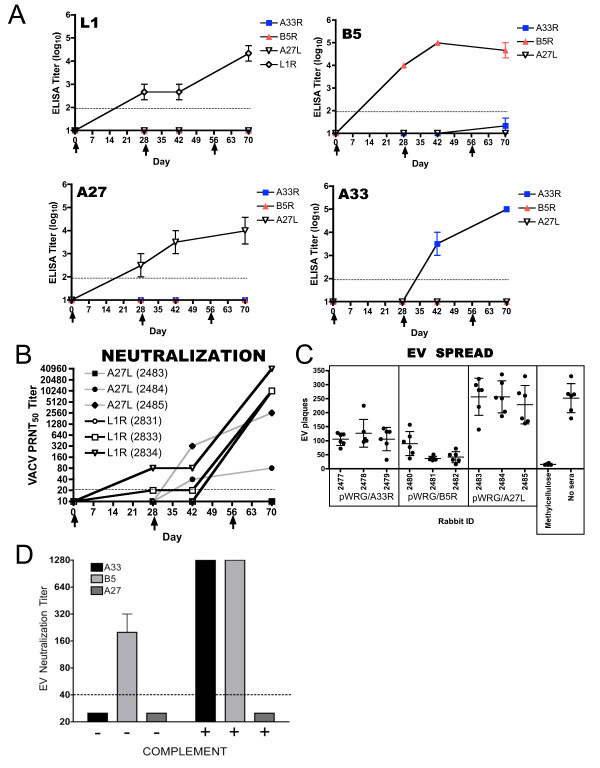
**Antibody responses after vaccination with individual poxvirus DNA vaccines administered using muscle electroporation**. A. Sera collected from three rabbits vaccinated with pWRG/A33R, or pWRG/B5R, or pWRG/A27L, or pWRG/TPA-L1R(opt) were tested for antibodies that bind A33, B5, A27, and L1 by immunogen-specific ELISA. Symbols represent mean endpoint ELISA titers ± SE. The limit of detection was a titer of 2 log_10 _(dashed line). **B**. Sera from rabbits vaccinated with the MV-specific targets, L1 and A27, were tested for VACV neutralizing antibodies by PRNT. Titers for each time-point are shown for individual rabbits. The limit of detection was a titer of 20 (dashed line). **C**. Day 70 sera from rabbits vaccinated with the EV-specific targets, A33 and B5, were tested for their capacity to prevent the spread of EV in an EV spread inhibition assay. Sera from rabbits vaccinated with pWRG/A27L were included as negative controls. Other controls included wells that were overlain with methylcellulose after the adsorption step (no EV spread) and well that received media without dilute sera (no sera control). Symbols represent sera diluted 1:14-1:56. Numbers of satellite plaques per well are shown as scatter graphs. The mean ± SD for each serum sample is shown. Rabbit ID # and the plasmid vaccine used to generate the sera are shown on the x-axis. **D**. Sera from rabbits were diluted and samples were incubated with fresh EV particles in the presence or absence of complement as indicated. EV neutraliation titers were determined as described in the material and methods. The limit of detection was a titer of 40 (dashed line).

To investigate the possibility of producing a protective immunogen-specific polyclonal antibody cocktail using DNA vaccine technology, we used individual DNA vaccine plasmids to produce polyclonal antibodies in separate rabbits. Rabbits were vaccinated three times by muscle electroporation on days 0, 28, and 56 with DNA vaccines encoding L1, A27, B5, or A33 proteins. Sera collected on days 0, 28, 42, and 70 were evaluated for anti-poxvirus antibodies by immunogen-specific ELISA, PRNT, and EV inhibition assay (Figure 2). ELISA results are shown in Figure 2A. All prevaccination (day 0) sera were negative for VACV-specific antibodies by ELISA. After a single vaccination, anti-L1, -A27, and -B5 antibodies were detected, while antibodies against A33 were not detected until after the second vaccination with the A33R DNA vaccine. After the third vaccination (day 70), high-titer antibodies against L1 (GMT = 4.3), A27 (GMT = 3.9), B5 (GMT = 4.6), and A33 (GMT = 5) were produced. PRNT results are shown in Figure 2B. As expected [28,30-34], only the A27L and L1R DNA vaccines elicited MV neutralizing antibodies. Two of the three rabbits vaccinated with L1R developed neutralizing antibodies after one vaccination, and all three developed titers > 1000 after the third vaccination. Only two of the three rabbits vaccinated with A27L developed neutralizing antibodies, both after the second vaccination. EV inhibition assay results are shown in Figure 2C. Sera from rabbits vaccinated with A33R, B5R, or A27L DNA vaccines were tested for a capacity to inhibit the spread of EV. EV spread was inhibited by sera from animals vaccinated with either B5R or A33R DNA vaccines, but not by the A27L vaccine (Figure 2C). The anti-B5 response was more effective than the anti-A33 responses at inhibiting EV spread. We also examined the ability of anti-EV antibodies to neutralize or disrupt EV particles in the presence or absence of complement + anti-L1 MAb-10F5. Sera from B5-vaccinated rabbits neutralized EV particles in the presence or absence of complement with titers of 180 and > 1280, respectively (Figure 2D). In contrast, anti-A33 sera only neutralized EVs in the presence of complement with a titer of > 1280. Sera from A27-vaccinated animals did not neutralize EVs. Together, these findings demonstrated that it was possible to use muscle electroporation for plasmid DNA vaccination to generate polyclonal antibodies that not only bound to L1, A27, A33, and B5, but also were functionally active; two of the vaccines (L1R and A27L) elicited MV neutralizing antibodies, and the other two vaccines (A33 and B5) elicited antibodies that inhibited EV spread in cell culture.

### Bioimaging of normal BALB/c mice infected with IHD-J-Luc recombinant vaccinia virus after prophylactic treatments with immune rabbit sera

**Figure 3:**
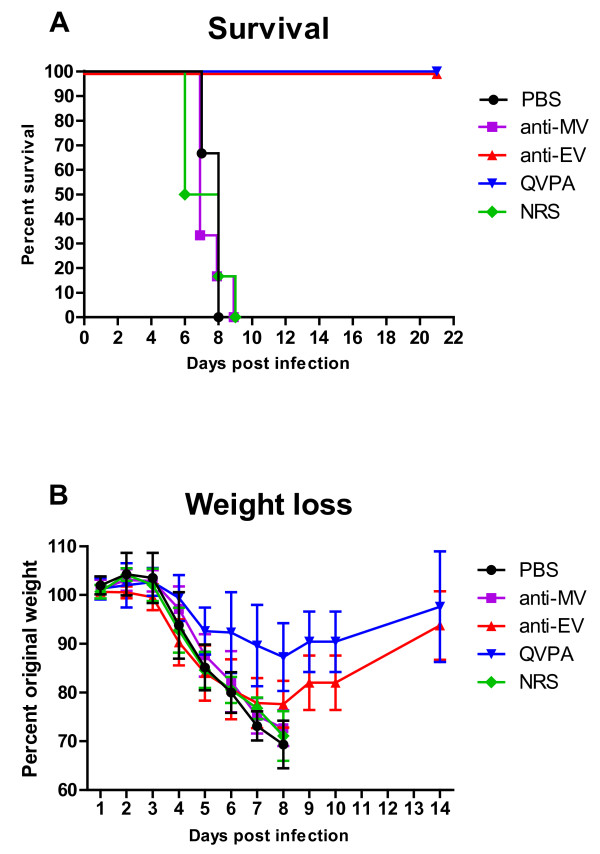
**Testing of rabbit sera cocktails against MV, EV, or a combination of MV and EV (QVPA) in a lethal IHD-J-Luc vaccinia virus intranasal challenge model**. BALB/c mice were inoculated with anti-MV (purple), anti-EV (red), or QVPA (blue) cocktails, or with normal rabbit sera, (NRS, green) one day before challenge with IHD-J-Luc; control mice received similar volumes of PBS and are shown in black. Mice were observed for lethality (**A**) and for weight loss (**B**) for 21 and 14 days, respectively. The anti-MV cocktail contained1:1 mixture of anti-L1 and anti-A27 sera. Anti-EV cocktail contained 1:1 mixture of anti-B5 and anti-A33 sera. QVPA (Lot 1) contains both anti-MV and anti-EV polyclonal sera and PBS as described in Material and Methods. The IHD-J-Luc control group, n = 9, all other groups, n = 6.

Having generated antibodies against L1, A27, B5 and A33, we next characterized the ability of these molecules to prevent viral dissemination and protect mice from lethal VACV infection when delivered as combinations against the EV and/or MV particles. Equal volumes of day 70 sera from rabbits vaccinated with the L1R, A27L, A33R, and B5R DNA vaccines were combined to make a quadrivalent polyclonal antibody (QVPA) cocktail at a 1:1:1:1 ratio (Table 1). QVPA was characterized by ELISA, PRNT, and an EV-spread assay to confirm binding and functional activity. ELISA titers (log_10_) against L1, A33, A27, and B5 were 4, 4, 5, and 5, respectively. The VACV PRNT_50 _titer was 3620. The QVPA reproducibly inhibited EV spread when diluted 1:100 (data not shown). Additionally, QVPA neutralized EV particles with an EV neutralization titer of 80. In the presence of complement the EV neutralization titer markedly increased to > 1280. Anti-MV (A27 and L1) and anti-EV (A33 and B5) targeting combinations where generated such that the amount of antibodies against each target were equimolar to that of the QVPA. Furthermore, to better understand the mechanisms by which QVPA, anti-MV, and anti-EV rabbit sera protect animals, we used whole-body bioimaging to follow virus replication and dissemination to the lungs and internal organs after intranasal inoculation of BALB/c mice with IHD-J-Luc recombinant VACV expressing luciferase [46,47]. Mice were inoculated with a single dose of the anti-MV, anti-EV, or QVPA cocktails, with NRS, or with PBS in control 1 day before intranasal challenge with 10^5 ^PFU of IHD-J-Luc and were followed for survival and weight loss (Figure 3). Control mice and mice that received NRS succumbed to death between days 7-8. QVPA sera protected 100% of mice from lethality, as well as morbidity as judged by significantly reduced weight loss on days 5, 6, and 7 compared with control animals (Figure 3A and 3B and Table 2). Anti-EV cocktail protected 100% of mice against IHD-J-Luc-induced lethality, yet it did not protect from weight loss (Figure 3A and 3B and Table 2). Anti-MV cocktail did not protect from lethality or from weight loss in the IHD-J-Luc challenge model (Figure 3).

**Table 1:** Polyclonal antibody cocktails used in studies

	Day 70 rabbit sera				
	
Cocktail	anti-L1R	anti-A27	anti-A33	anti-B5	ratio
QVPA	#2834	#2485	#2478	#2482	1:1:1:1
anti-MV	#2834	#2485	-	-	1:1
anti-EV	-	-	#2478	#2482	1:1
NRS	#1339	-	-	-	NA

NRS = normal rabbit sera

NA = not applicable

**Table 2:** Mean weight change in mice challenged with VACV IHD-J following administration of rabbit sera

	Percent weight change		
**Treatment**	**Day 5**	**Day 6**	**Day 7**

PBS	-14.8 ± 4.7	-20.0 ± 4.2	-26.8 ± 3.0
Anti-MV	-12.4 ± 4.3	-17.8 ± 6.3	-24.8 ± 3.6
Anti-EV	-16.0 ± 5.7	-19.3 ± 6.2	-22.2 ± 5.1
Anti-QVPA	-7.4 ± 4.8*	-7.7 ± 8.3*	-10.3 ± 8.3**
NRS	-15.4 ± 3.7	-19.5 ± 2.7	-23.3 ± 2.2

**Figure 4:**
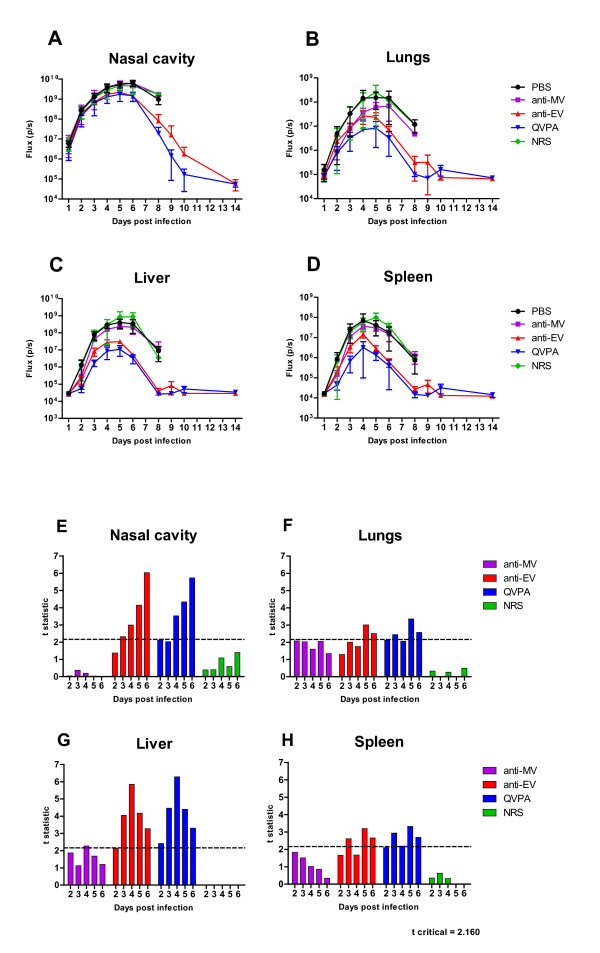
**Bioluminescence in the organs of mice pretreated with rabbit sera before infection with IHD-J-Luc**. Mice were inoculated s. c. with anti-MV (purple), anti-EV (red) cocktails, or with QVPA (blue), NRS (green), or PBS (black) on day -1 and were infected with IHD-J-Luc VACV on day 0. Animals were subjected to whole-body imaging daily for 10 days. Bioluminescence in the nasal cavity (**A, E**), lungs (**B, F**), liver **C, G**), and spleen (**D, H**) was recorded and used to calculate mean total fluxes ± SD (**A-D**) and t-statistic (**E-H**). Axis "y" in panels E-H shows the value of t; t ≥ 2.16 is significant at α = 0.05 (two-tailed) for groups of 6 mice; t = 2.16 is depicted with broken horizontal line (**E-H**).

All mice were subjected to bioimaging daily for 10 days using IVIS 50 instrument and bioluminescence recorded in the nasal cavity, lungs, liver, and spleen was used to calculate mean total fluxes ± SD (Figure 4A-D). Representative images collected on sequential days from control group and from QVPA-treated group exhibiting bioluminescence in the nasal cavity, lungs, spleen, and liver are shown in the Additional File 1. Background bioluminescence in internal organs was measured in BALB/c mice before infection and the mean total fluxes were around 10^4 ^photons/second (p/s) in the nasal cavity, liver, and spleen and 10^5 ^p/s in the lungs (Data not shown). At 24 h postinfection with IHD-J-Luc, all mice exhibited bioluminescence signal in the nasal cavity that was on average two logs above background (Figure 4A). In PBS-treated mice and in mice that received NRS or anti-MV cocktail, mean total fluxes increased rapidly within first 4 days postinfection and were maintained at high levels until mice succumbed to death (Figure 4A, Additional File 1A, and data not shown). In the nasal cavities of QVPA and of anti-EV pretreated mice, viral replication increased between days 2-6 and then returned to background levels by day 10-14 (Figure 4A). None of the immune rabbit sera prevented initial dissemination of IHD-J-Luc from the site of inoculation to internal organs (lungs, liver, or spleen) (Figure 4B, C and 4D). Yet clear differences between treatment groups were noted. In mice that received QVPA and anti-EV cocktails, the mean total fluxes within the internal organs increased during days 1-4, reached plateau on days 4-6, and then dropped to background levels on day 8 (Figure 4B-D blue and red lines). In contrast, mice that received anti-MV cocktail showed higher signals throughout the observation period and the curves were indistinguishable from the NRS control group. Mean total fluxes were 2 logs higher in mice from MV- and from NRS-treated groups that survived by day 8 compared with mice that received QVPA or anti-EV cocktail (Figure 4B-D).

To determine if differences in total fluxes between mice that received rabbit sera and control mice were significant, we subjected total fluxes recorded in individual mice between days 2 and 6 to t statistic (Figure 4E-H). All values above the horizontal lines represent statistically significant differences between total fluxes of a given treatment group compared with PBS-treated control animals. The mean total fluxes in the nasal cavity were significantly different between mice that received anti-EV or QVPA cocktails and control mice on days 3-6 or 4-6, respectively (Figure 4E red and blue bars). In the lungs, anti-EV and QVPA significantly reduced mean total fluxes on days 5 and 6 and on days 3, 5, and 6, respectively. In the liver, the significant differences for the same groups were observed on days 2-6 (Figure 4G), and in the spleen on days 3, 5, and 6 and days 3-6, respectively (Figure 3F and 3H). NRS and anti-MV cocktail did not significantly reduce mean total fluxes in any organ (Figure 4E-H, green and purple bars).

Altogether these data showed that QVPA and anti-EV cocktails, but not anti-MV cocktail, protected animals from lethality and significantly reduced viral replication at the site of inoculation, as well as in the lungs, spleen, and liver. Importantly, the reduction in viral replications was observed as early as day 3 in the nasal cavity, liver, and spleen, and was sustained for the following days. Thus, bioimaging allowed us to confirm that effective protection from lethality by rabbit immune sera correlated with early (day 3-4) reduction of viral loads in key organs.

### Reduction in pox formation after pretreatment with antibody cocktails

**Figure 5:**
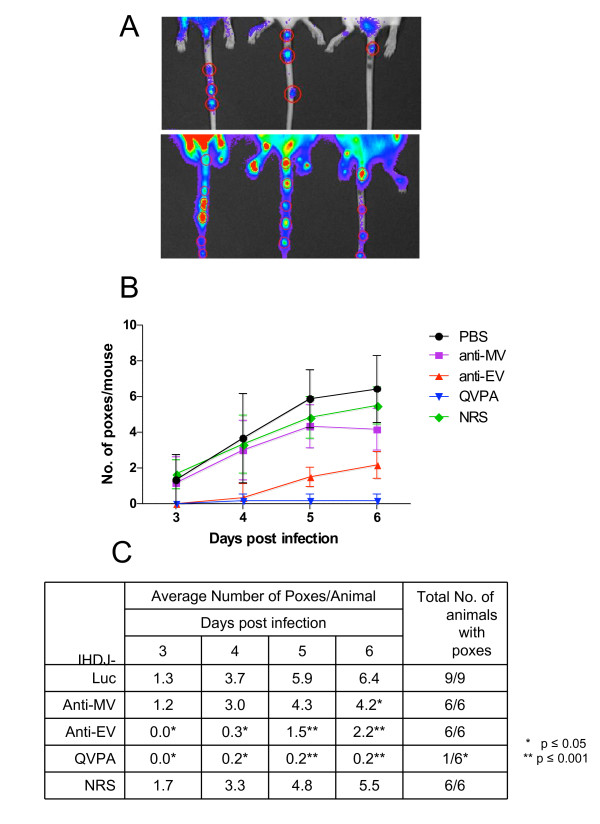
**Effect of anti-MV and anti-EV rabbit sera on pox development in mice challenged with IHD-J-Luc**. IHD-J-Luc challenged mice described in the legend to Figure 4 were subjected to whole-body imaging. The representative images of tails of three mice from the control group (PBS treatment) recorded on day 3 (top panel) and day 5 (lower panel) are shown (**A**). Pox lesions were scored using sequential daily images and used to calculate mean number of pox lesions per animal per day ± SD on days 3-6 (**B, C**) and total number of animals that developed pox lesions during observation period (**C**).

We next examined the ability of immune rabbit sera to prevent pox formation in the tails of infected mice. Pox lesions were scored as bioluminescence foci on the tails of each animal on days 3-6 postchallenge (Figure 5A). All control animals, animals that received NRS, or anti-MV cocktail generated pox lesions (Figure 5B and 5C). QVPA cocktail both dramatically reduced the numbers of mice with pox lesions and the number of pox lesions per animal (Figure 5B and 5C). All mice that received anti-EV cocktail developed pox lesions but the numbers of lesions per animal were significantly lower than in control group (Figure 5B and 5C). These data showed that QVPA cocktail that contains antibodies against both EV and MV viral isoforms was the most efficient in preventing pox lesion development. The anti-EV antibody cocktail curtailed virus dissemination to internal organs (Figure 4), but did not completely prevent pox lesion formation. Anti-MV antibodies did not affect pox lesions on their own, but did contribute to protection from pox lesion development when combined with the anti EV antibodies in the QVPA cocktail

### QVPA protects SCID mice from lethal disease

**Figure 6:**
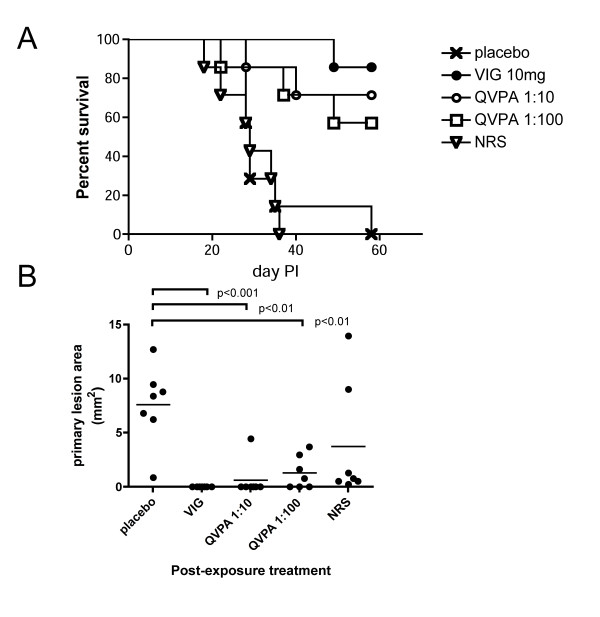
**Efficacy of immune rabbit sera in a mouse model of progressive vaccinia**. Groups of 6-week old SCID/NCr mice (n = 7/group) were scarified with VACV, then treated i.p. with PBS (placebo), VIGIV (10 mg), QVPA (1:10; 1:100), or NRS (1:10) on days 2, 5, 10, and 15. (**A**) Survival was monitored out to 60 days postinfection. VIGIV and both QVPA dose groups showed a statistically significant increase (p < 0.05) in median survival time compared to placebo or NRS. (**B**) Primary lesion size on day 19 was determined from digital photographs and compared using one way ANOVA and Tukey's multiple comparison. Treatment with VIGIV or either dose of QVPA resulted in statistically significant reduction (p < 0.01) in lesion area.

Scarification of severely immunodeficient mice (SCID) results in expanding lesions at the vaccination site in a manner that closely resembles human progressive vaccinia, a severe life-threatening complication observed with live-virus vaccinia vaccines [48]. We utilized this model to test the protective efficacy of QVPA. Mice were treated with 0.2 mL of placebo (PBS), 10 mg of VIGIV, 0.2 mL of QVPA at 1:10 or 1:100 dilutions in PBS, or 0.2 mL of NRS at 1:10 in PBS on days 2, 5, 10, and 15. The VIGIV used in this experiment had a PRNT_50 _titer = 453. ELISA titers (log_10_) against L1, A33, A27, and B5 were 2, 3.5, 3, and 4, respectively. We previously demonstrated that this aggressive VIGIV therapeutic regimen protects immunocompromised mice against progressive vaccinia [49]. Here, survival in the VIGIV treatment group was 85%, with 0% of placebo mice surviving. QVPA was also effective in preventing death in infected mice under identical treatment conditions, although a dose response was not observed (Figure 6A). As expected, VIGIV treatment resulted in lesion regression in all mice by day 19. QVPA at either 1:10 or 1:100 also resulted in lesion regression in most mice and correlated with survival (Figure 6B). Several mice receiving NRS also resolved the primary lesion, although they succumbed to VACV infection in a manner similar to placebo treated mice.

## Discussion

### Polyclonal antibodies targeting both the *Orthopoxvirus *MVs and EVs are critical for protection

VIGIV is known to contain antibodies that bind proteins on both the MV and EV [22,50,51]. More importantly, this polyclonal product contains antibodies that neutralize both MV and EV. Although it is not clear which antibody subset in VIGIV is involved in protective immunity, it is likely both subsets contribute to protection. Our group found [30,31,34], and it was later independently confirmed [24], that combinations of antigens targeting the two particles types of orthopoxviruses provided superior protection in vaccinated animals. Similarly, Lutsig et al, demonstrated that monoclonal and polyclonal antibody combinations targeting these molecules provided enhanced protection compared to targeting individual viral molecules [52]. Here, we expanded these studies and analyzed the impact of polyclonal antibodies targeting EV and MV particles individually or both EV/MV particles together (QVPA). Using bioluminescent virus, we observed several differences in the ability of anti-EV and anti-MV antibodies to protect mice from VACV IHD-J. Anti-MV antibodies in immune sera failed to provide protection against VACV IHD-J, whereas antibodies against EV provided protection from lethality and significantly reduced viral replication at the site of inoculation (nasal cavity) and reduced dissemination and/or replication in the lungs, spleen, and liver. QVPA was most efficient in protecting from lethality, and dissemination, and unlike anti-EV cocktail, also significantly reduced weight loss. In addition, QVPA significantly reduced numbers of pox lesions in surviving mice. Thus, of the three tested combinations, the EV and QVPA were similarly efficient in preventing lethality, yet only QVPA also protected from morbidity and pox lesion formation, suggesting that both anti-EV and anti-MV antibodies are required for complete protection.

The precise mechanism(s) by which anti-EV and anti-MV antibodies mediated protection was beyond the scope of this investigation. However, several mechanisms, none mutually exclusive, have been proposed. Data from several studies indicate that anti-MV antibodies mediate protection by neutralizing MV during initial target cell interactions (e.g., attachment and penetration) and by neutralizing MV released from lysed infected cells or disrupted EV [53-58], and Schmaljohn A., personal communication). Anti-EV antibody mediated protection appears to be more complex and likely involves multiple mechanisms [26,59,60]. These may include the ability of anti-EV antibodies to prevent viral egress [60], thereby preventing EV spread *in vivo*, or to prevent EV binding and entry [26]. Recently, using anti-B5 monoclonal antibodies, a role for complement in protection was reported by the Crotty group [61]. Lustig et al, produced some evidence to suggest a synergistic protective role of anti-EV and anti-MV antibodies [58]. In this model, anti-EV antibodies play a role in complement-mediated disruption of the EVs, followed by MV neutralization by anti-MV targeting antibodies. This may explain why combinations of antibodies targeting both particle types are more effective at protection compared to individually targeting each particle. We showed that both anti-A33 and anti-B5 antibodies are capable of disrupting EV particles in the presence of complement (Figure 2D), making the resultant MVs subject to neutralization by anti-MV antibodies. Interestingly, contrary to anti-B5 sera, anti-A33 antibodies did not neutralize EV in the absence of complement. These findings suggest that anti-A33 protection is predominantly mediated by complement, whereas anti-B5 mediated protection can be complement dependent or independent. In the experiments described in this report, rabbit antibodies were used in a murine system. The capacity of rabbit antibodies to fix murine complement is suboptimal [62]. Thus, it is likely that complement did not play as much of a role in the observed protection that it would in a homologous system. Regardless of mechanism, our findings clearly demonstrate that addition of both EV and MV targeting antibodies resulted in superior protection.

### Antibodies against each antigen contribute to protection

**Figure 7:**
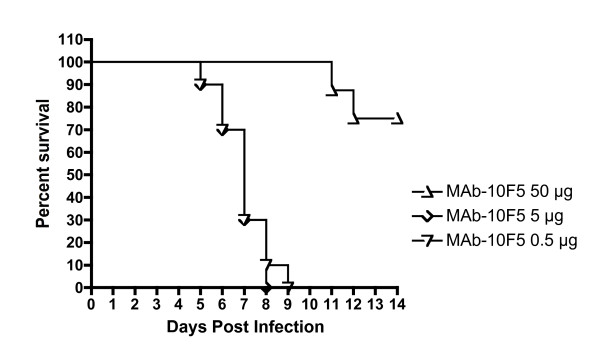
**Titration of protection conferred by passive transfer of anti-L1 monoclonal antibody in VACV IHD-J mouse model**. Groups of seven (50 μg dose) to 10 (5 and 0.5 μg groups) mice were inoculated s. c. with purified anti-L1 MAb-10F5 diluted in PBS. The next day, all 27 mice were challenged with 2 × 10^6 ^PFUPFU of virus intransally. Mice were monitored for 20 days. Moribund animals, or animals that lost more than 30% of their starting weight, were euthanized. Days 0-14 are shown. No additional animals succumbed after day 12.

The polyclonal anti-MV antibodies alone did not confer significant protection in the VACV IHD-J i.n. challenge. However, as shown in Figure 1, it is possible to protect mice in the intranasal VACV IHD-J challenge model using high concentrations of anti-MV monoclonal antibodies. In that experiment, anti-L1 monoclonal antibodies, MAb-7d11 or MAb-10F5, at a 100 μg/mouse dose were protective. Both anti-L1 MAbs used in the experiment had a 50% neutralization titer of ~10 ng/ml. This value is the same that was reported for MAb-7D11 by Wolfe et al. [54]. Thus, the 100 μg dose was equivalent to 10,000 neutralizing units per mouse. A dose-ranging study using MAb-10F5 in the VACV IHD-J intranasal model demonstrated that the protective effect was diminished to 80% when 5,000 neutralizing units/mouse were delivered, and undetectable when 500 neutralizing units were delivered (Figure 7). The anti-MV titer in QVPA PRNT_50 _titer = 3620. Thus, the 0.3 ml dose delivered had only 1086 neutralizing units per mouse; a dose insufficient for significant protection in the absence of anti-EV antibody.

The polyclonal anti-EV component alone conferred a significant level of protection in the VACV IHD-J mouse model. The anti-A33 and anti-B5 antibodies within this cocktail were capable of preventing the spread of EV in cell culture (Figure 2) suggesting the antibodies were conferring protection by binding EV and inhibiting spread *in vivo*. Our data demonstrate that there was less spread within the nasal cavity, less spread to the liver, lungs, and spleen, and even less spread as measured by tail pox lesions. It is possible that the anti-EV antibodies within the cocktail are also involved in destruction of infected cells or other means of infection clearance. The more rapid clearance of virus from the nasal cavity, lungs, liver, and spleen (Figure 4) when anti-EV antibodies were present support this possibility; however, at this time we do not have any direct evidence that antibodies in the QVPA are involved in antibody-dependent cell-mediated cytotoxicity (ADCC) or complement-dependent cytotoxicity (CDC).

### An alternative to VIGIV

VIGIV is an integral component of a multilayer strategy to defend against orthopoxviruses, in particular as it relates to biodefense preparedness. In recent years it has been used in combination with other experimental drugs to treat serious adverse events associated with vaccination with the smallpox vaccine. For example, in 2009 VIGIV was used to treat a military smallpox vaccinee with progressive vaccinia [48]. In addition to its value in treating vaccine adverse reactions, it is also likely that VIG would be considered for use as an emergency prophylactic or therapeutic during an *Orthopoxvirus *outbreak, such as the North American MPXV outbreak in 2003 [63]. Despite its importance, the precise anti-vaccinia antibody composition of VIGIV and the mode of action of this drug are poorly understood. As is the case with all plasma-derived products, despite donor screening, plasma testing, and viral inactivation steps used during manufacture, there remains the risk of transmission of blood-borne viral or prion disease. Immune globulin intravenous (human) products have also been associated with various adverse events such as renal dysfunction, hypersensitivity reactions, and thrombotic events [64]. Moreover, the amount of VIGIV needed to treat adverse events might be underestimated, as is its interaction with other co-administered therapeutics. In the 2009 progressive vaccinia case, a total of 16,740,000 units of intravenous VIGIV were administered during over 3 months, along with oral and topical ST-246, CMX001, and topical imiquimod. The total amount of product administered was more than 334 doses, an amount previously thought sufficient for up to 30 patients [48]. These factors have led health officials to suggest replacing VIGIV with a new product with high-specific activity, well defined antibody specificity and a similar or improved safety profile [12,13]. Potential VIGIV-replacements include human monoclonal antibodies [47,65], humanized monoclonal antibodies [66], and chimpanzee anti-B5 and anti-A33 monoclonal antibodies [67,68].

The 4pox DNA vaccine targets immunogens located on the MV (L1 and A27) and EV (A33 and B5). This vaccine establishes protective immunity against orthopoxviruses as evidenced in several *Orthopoxvirus *challenge models, including MPXV challenge of NHPs [32,34] and Golden, J.W. et al manuscript forthcoming). The current study demonstrates that the antibodies produced by this vaccine are sufficient for protection, even in immunocompromised mice (Figure 6). Despite being a crude serum preparation, QVPA provided significant protection against progressive vaccinia in the mouse model. The level of protection was comparable to that provided by a total dose of 40 mg of VIGIV, which is a purified IgG preparation, suggesting that a combination of specific MV and EV components may result in a treatment with high specific activity. There could be some non-specific inhibitory effects associated with the introduction of heterologous (rabbit) sera into mice because several animals treated with NRS had reduced lesion size compared to placebo but, nevertheless, still demonstrated a similar median survival time compared to placebo controls. The ability of our 4pox DNA vaccine platform to generate highly potent protective polyclonal antibodies against defined and protective *Orthopoxvirus *targets argues this system could be a suitable replacement for VIGIV that was produced from pooled plasma from Dryvax-vaccinated individuals. QVPA offers an advantage over monoclonal mixtures, another possible VIGIV replacement, because monoclonal antibody escape mutants have been shown to exist among poxviruses. For example, a single-point mutation in L1 at amino acid position 35 can prevent viral neutralization by some otherwise neutralizing antibodies [53]. This is a result of changes in the tertiary structure of the previously reported L1 neutralizing epitope [69]. Accordingly, polyclonal antibodies may be preferable for new immunoglobulin product seeking to mitigate the deleterious events associated with a bioterrorism event involving *Orthopoxvirus*, in particular genetically modified viruses. Additionally, redundant targeting of the EV particles will be critical. We have reported that antibodies against A33 do not efficiently cross-interact with orthologs from VACV and MPXV [27]. Furthermore, others have reported similar cross-reactively problems associated with VACV and VARV B5 [70]. Therefore, any future immunotherapeutic should target both molecules, as well as the MV to ensure adequate cross-protection.

### Genetic vaccines for immunotherapeutic production

There is an emerging concern that some hostile entities may deploy biological weapons (BW) as weapons of mass destruction [71-73]. This concern is augmented by official United States Government reports expressing fear that BW technology is much cheaper and easier to obtain compared to nuclear, and yet can deliver significant destruction [74]. From a broader standpoint, the use of DNA vaccination technology as a platform for establishing protective immunity against bioterrorism threat agents is particularly alluring because it has the potential for rapid development of scalable vaccines (months versus years) that are human-safe. This rapid development is facilitated by the fact that molecular vaccines consist of DNA, which is obtainable by the advent of sophisticated genomic sequencing, even without the need to isolate an organism (e.g., sequencing the pathogen genome from infected tissue and de novo synthesis of desired target, such as glycoproteins) [75]. DNA can be produced under good-manufacturing product specifications (GMP) without the need for complex and time-consuming processes of growing/attenuating/killing virus or protein purification [76,77]. The data presented in this study suggest another benefit of this technology could be the relatively rapid generation of protective antibodies. These antibodies could be used to aid in protection against threat agents whose release into the environment is imminent or occurring. These antibodies could also be utilized in diagnostic tests. Moreover, the antibodies could be used as immunotherapeutics to bridge the immunity of persons exposed to threats while vaccine-induced immunity builds. It could also enhance and speed up adaptive immune responses through the rapid generation of immune complexes in vivo [78]. This is similar to postexposure treatment of rabies, where anti-rabies antibodies are co-administrated with the vaccine [79]. DNA vaccine technology would seem particularly useful for protection strategies involving the simultaneous use of antibodies and vaccine, as the efficacy of gene-based vaccines are not reduced by neutralizing antibodies whereas the efficacy of whole virus containing vaccine can be impacted by the presence of antibodies. The notion of using DNA vaccines to develop human-safe immunotherapeutics is augmented by the development of humanized recombinant animal systems that possess the ability to generate human antibodies, including cattle capable of generating nearly-human antibodies [80] and by direct cloning of human heavy and light Ig chains from antigen-specific B cells after smallpox vaccination (55). Such antibodies would circumvent potential reactogenicity of antibodies made in other species. The extent by which DNA vaccine technology can be used to develop immunotherapeutics against infectious agents, including biological threat agents for which neither immune sera produced using licensed vaccines nor convalescent sera/plasma are available, is a matter for future exploration.

## Materials and methods

### Animals

Female BALB/c and SCID/NCr mice were obtained from the National Cancer Institute and used for passive transfer experiments. Female New Zealand white rabbits were used for the generation of rabbit PAbs by DNA vaccination. All animal work was approved by institutional animal care and use committees.

### Cells and viruses

VACV Connaught vaccine strain (derived from the New York City Board of Health strain), VACV strain WR (ATCC VR-1354), and VACV strain IHD-J (obtained from Dr. Alan Schmaljohn) were all maintained in VERO cell (ATCC CRL-1587) monolayers grown in Eagle minimal essential medium, containing 5% heat-inactivated fetal bovine serum (FBS), 1% antibiotics (100 U/ml penicillin, 100 μg/ml of streptomycin, and 50 μg/ml of gentamicin), 10 mM HEPEs (cEMEM). A HeLa cell passage of the smallpox vaccine virus (Dryvax; derived from the New York City Board of Health strain and produced as a vaccine by Wyeth) was obtained from the laboratory of Dr. Michael Merchlinsky via Hana Golding (CBER, FDA). COS-7 (COS) cells (ATCC CRL-1651) were used for transient expression experiments. BSC-1 cells (ATCC CCL-26) were used for plaque-reduction neutralization assays (PRNT) and EV spread assays. Both BSC-1 and COS-7 cells were also maintained in cEMEM.

### DNA vaccination of rabbits by electroporation

Rabbits were vaccinated using muscle electroporation as described previously [81]. Briefly, acclimated female New Zealand white rabbits were vaccinated with 1.3 mg of plasmid DNA per vaccination per animal using an Inovio (San Diego, CA) Twin Injector electroporation device. Rabbits were vaccinated on days 0, 28, 56, and sera for QVPA were collected on day 70.

### Immunogen-specific ELISA

VACV histidine-tagged antigens L1 (300 ng/well), A33 (50 ng/well), B5 (50 ng/well) and A27 (50 ng/well), produced in *Escherichia coli *or mammalian cells (B5 antigen produced in baby hamster kidney cells) and purified on nickel columns, were diluted in 0.1 M carbonate buffer [pH 9.6] and plated in duplicate in the wells of a high-binding, 96-well plate (Corning; Corning, NY). The ELISA was performed in an identical manner as previously described (40). For assays involving anti-pox mouse antibodies, the secondary antibody was hydrogen peroxidase conjugated goat anti-mouse Ig (Sigma Cat no. A4416) diluted 1:1000; and for assays involving anti-pox rabbit antibodies, the secondary antibody was hydrogen peroxidase conjugated goat anti-rabbit Ig (KPL Cat no. 04-15-06) diluted 1:1000. For assays involving VIGIV, the secondary antibody was hydrogen peroxidase conjugated goat anti-human IgG, A, M (KPL Cat no. 074-1007) diluted 1:500. End-point-titers were determined as the highest dilution with an absorbance value greater than the mean absorbance value from at least three normal sera plus three standard deviations.

### Neutralization Titers

The plaque-reduction neutralization test (PRNT) involving VACV strain IHD-J and BSC-1 cells was described previously (43). All sera tested in the PRNT were incubated at 56°C for 30 min. Plaques were counted and the percent neutralization was calculated relative to the number of plaques in the absence of antibody. Titers represent the reciprocal of the highest dilution resulting in a 50% reduction in the number of plaques. Mean neutralization titers for individual mice were plotted ± standard deviation.

### Anti-EV functional antibody assays

The EV spread inhibition assay was performed as follows. VACV strain IHD-J was diluted in cEMEM to give ~25 PFU/ml. Aliquots of this viral suspension (100 μl) were adsorbed onto BSC-1 confluent cell monolayer in 6-well plates for 1 h in a 37°C 5% CO_2 _incubator. Plates were rocked ~15 min. After adsorption cells were rinsed once with cEMEM and sera from the indicated groups was added to the wells. All serum samples were heat activated at 56°C for 30 min prior to use. After 18 h in a 2 ml semisolid overlay (Earle's basal minimal essential medium, 1.5% methyl cellulose, 5% heat-inactivated FBS, supplemented with antibiotics (100 U/ml penicillin, 100 μg/ml of streptomycin, and 50 μg/ml of gentamicin)) was added to each well. The cells were incubated for 3 d at 37°C in a 5% CO_2 _incubator. On day 3, cell monolayers were stained with 1 ml of a staining solution (3% crystal violet and 15% ethanol in H_2_O). Satellite plaques were counted and the percent spread inhibition was calculated relative to the number of plaques in the absence of antibody. All samples were analyzed in duplicate.

The EV neutralization assay was preformed as reported previously [66]. Briefly, fresh EV particles (75-100 PFU) were incubated for 1 h with the indicate sera (diluted twofold starting at a 1:40) in the presence or absence of 5% baby rabbit complement (Cedarlane) in 200 μL total volume. After incubation, 180 μl of the mixture was adsorbed for 1 h on BSC-1 cell monolayers in 6-well plates. Warm PBS was used to wash away unbound virus and 2 ml semisolid overlay (see above) was added to each well. All samples included an anti-MV antibodies, MAb-10F5 (1:100) which targets the L1 molecule [30]. After 4 days, plaques were stained with crystal violet as described above. Plaques were counted and the percent neutralization was calculated relative to the number of plaques in the absence of anti-EV antibody or in the absence of anti-EV antibody, but in the presence of complement. EV neutralization titers represent the reciprocal of the highest dilution resulting in a 50% reduction in the number of plaques. Mean neutralization titers for individual mice were plotted ± standard deviation.

### Passive transfer of antibodies

For experiments using non-luciferase expressing VACV, Groups of eight BALB/c mice (9-11-weeks old) were anesthetized, ear tagged, weighed, and bled on day -7. On day -1 the mice were weighed again and then 300 μL of the indicated heat-inactivated rabbit or NHP serum from a previous study [34] were injected subcutaneously behind the neck. For combinations of rabbit sera, 75 μL of each PAb were administered in a total volume 300 μL. For monoclonal antibodies, the indicated concentration of MAb was diluted in PBS in a total volume of 300 μL and injected subcutaneously behind the neck.

### VACV strain IHD-J challenges

For experiments using non-luciferase expressing VACV, mice were anesthetized and weighed before intranasal administration of 50 μl of PBS containing 2 × 10^6 ^PFU of VACV strain IHD-J using a plastic pipette tip (25 μl per nare). This dose is three times the LD_50_. Subsequently, mice were observed and weighed daily for 14 d. Moribund mice (> 30% body weight) were euthanized.

### Progressive vaccinia model

Aliquots of HeLa-passaged Dryvax (VACV, New York Board of Health strain) were removed from -70°C storage, thawed on ice, and sonicated for three cycles each consisting of 15 sec at 90% power, 50% duty cycle and 15 sec on ice. Virus was diluted to the required concentration in cDMEM (Dubelco's minimal essential medium containing 10% fetal bovine serum, 4.5 mg/mL of D-glucose, 110 μg/mL of sodium pyruvate, 100 μM nonessential amino acids, 100 U/mL of penicillin/streptomycin, and 0.25 μg/mL of amphotericin) and kept on ice before scarification. To perform scarification, mice (n = 7 per group) were anesthetized with ketamine/xylazine and the fur removed from the lower back area using clippers. Ten μl of the virus inoculum (i.e., 10^6 ^PFU/mL for a 10^4 ^PFU challenge) were pipetted onto the skin covering the cartilageous area proximal to the base of the tail. A bifurcated vaccination needle (Precision Medical Products, Denver, PA) was used to deliver 20 punctures through the inoculums solution. VIGIV (10 mg), QVPA (1:10 or 1:100 in PBS), or normal rabbit sera (NRS) (1:10 in PBS) was administered via intraperitoneal (i.p.) injection on days 2, 5, 10, and 15 after VACV exposure. Mice were monitored for clinical signs including weight loss, ruffled fur, breathing difficulties, or hunched posture. Mice that lost 25% of their original body weight, exhibited limping or guarding behavior of a limb, remained hunched after stimulation, had difficulty breathing, had severe swelling of the mouth or throat interfering with eating or drinking, or were moribund were euthanized according to preset euthanasia criteria.

### Method for measuring lesion size

Lesion area analysis was performed with ImageJ using digital photographs and a calibrated reference as described in Rasband, W.S., ImageJ, U. S. National Institutes of Health, Bethesda, Maryland, USA, http://imagej.nih.gov/ij/, 1997-2011.

### Whole-body bioimaging using luciferase expressing VACV

A recombinant IHD-J vaccinia virus expressing luciferase (IHD-J-Luc) was constructed by Dr. Jerry Weir and Michael Merchlinsky and kindly provided for this study (81). Five-week-old female BALB/c mice (National Cancer Institute, Frederick, MD) were used in bioimaging experiments. Mice were anesthetized before infection by i.p. injection of 20 μl per g of body weight of 2, 2, 2-tribromoethanol dissolved in tertiary amyl alcohol and diluted in sterile PBS according to manufacturer's instructions. Mice were challenged i.n. with 10^5 ^PFU (2 LD_50_) of IHD-J-Luc in 10 μl volume delivered in one nostril.

Animals received a single dose administration of PBS, NRS, anti-EV, anti-MV, or QVPA cocktails in 400 μl volumes delivered via s. c. injections at the upper part of the back 1 day before challenge. The anti-EV and anti-MV cocktails were diluted 1:1 with PBS before injection to maintain similar concentration of anti-EV and anti-MV antibodies as in QVPA, The handling of mice and experimental procedures used in bioimaging were approved by the CBER animal study review committee

### In vivo measurements of luciferase activity

Ten to 15 min before imaging, mice received a single injection of D-luciferin potassium salt (Caliper Life Sciences, Hopkinton, MA) at 150 μg/g body weight, i. p. After anesthesia in the oxygen-rich induction chamber with 2% isofluorane, mice were imaged using IVIS 50 cooled charge-coupled device camera system (Caliper) as previously described [46]. Images were collected daily during first 10 days postchallenge, and were analyzed with the LivingImage 3.02 software (Caliper). The amount of light emitted by replicating virus in live animals was measured by establishing a single region of interest (ROI) for each organ as recommended by the manufacturer. The background bioluminescence was determined using images of D-luciferin-injected animals 1 day before infections and was subtracted from experimental values.

### Statistical analysis

Kaplan Meier survival curves of time-to-death after infection were generated using standard GraphPad Prism V5 software. Total lesion areas on day 19 were compared using one-way ANOVA and Tukey's multiple comparison test. t-statistic was employed to compare mean total fluxes between infected control mice (PBS-treated) and infected and treated mice (anti-EV, anti-MV, QVPA, and NRS) using Microsoft Excel.

## Competing interests

J. Hooper is a co-inventor on U.S. patents 6,562,376 and 6,620,412 that are indirectly related to the work described herein.

## Authors' contributions

JH and JG were involved in the concept and design of the experiments characterizing the polyclonal sera and using it in passive transfer studies. MZ, SK, and HG were involved in the concept and design of the challenge experiments involving bioluminescence. RF and MM were involved in the concept and design of the progressive vaccinia model work, and JB was in involved in the concept and design of the experiments involving muscle electroporation of rabbits for production of polyclonal sera. All authors were involved in the writing, editing, and reviewing the manuscript.

## Supplementary Material

Additional file 1 **Images of mice infected with IHD-J-Luc and either treated or not with anti-QVPA cocktail**. Mice described in the legend to Figure 4 were subjected to whole-body imaging daily for 10 days or during survival. Representative sequential images of mice 1, 2, and 3 from control group (PBS only, panels A and C) and of mice 1, 2, and 3 from the group that received QVPA (panels B and D) are shown. Images of heads (A, B) were acquired from the same mice as images of the torso (C, D) but using shorter exposure to avoid saturation of the camera due to high signal from the nasal cavity. Images from control and from treated mice were collected on day 1-6 (A, C) and on day 1-9 (B, D), respectively. Click here for file
