# Supplementary figures and images for: Polyclonal antibody cocktails generated using DNA vaccine technology protect in murine models of orthopoxvirus disease
Source: Virol J. 2011 Sep 20;8:441. doi: 10.1186/1743-422X-8-441 (PMC3192780; doi:10.1186/1743-422X-8-441)

A

D 1-3

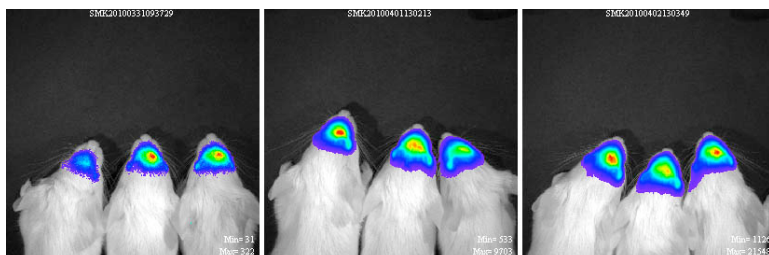

D 4-6

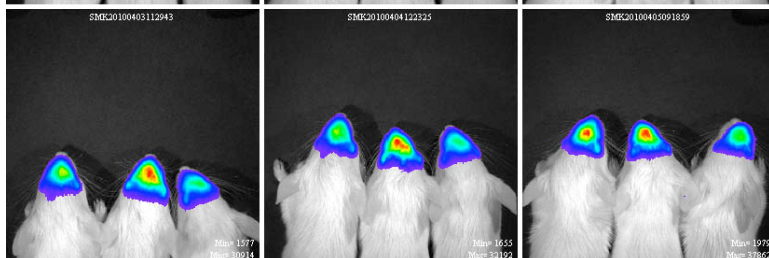

B

D 1-3

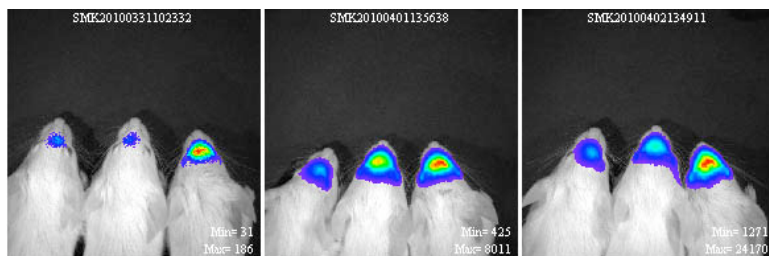

D 4-6

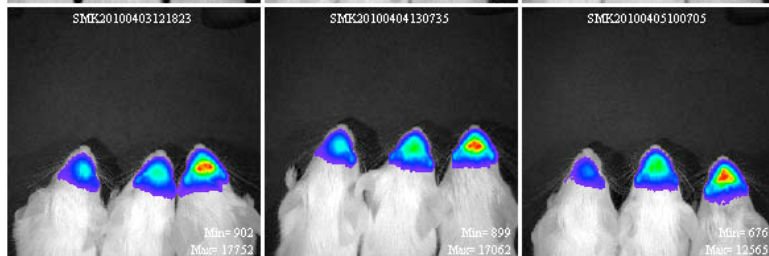

D 7-9

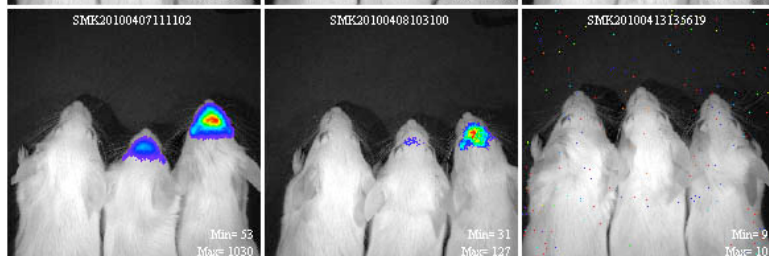

C

D 1-3

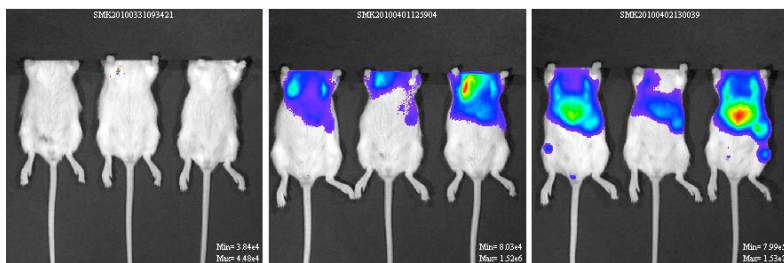

D 4-6

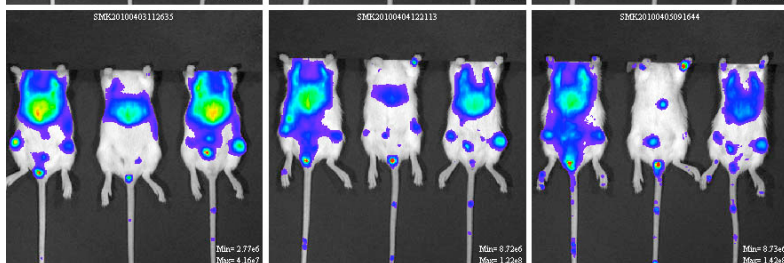

D

D 1-3

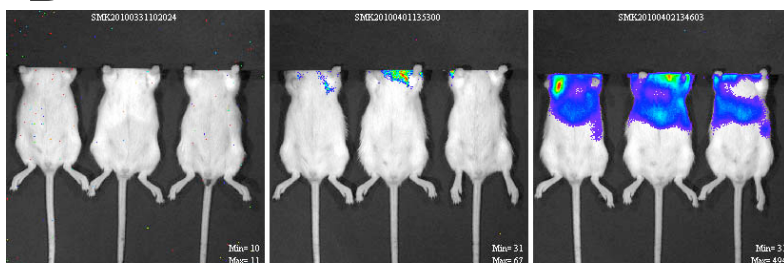

D 4-6

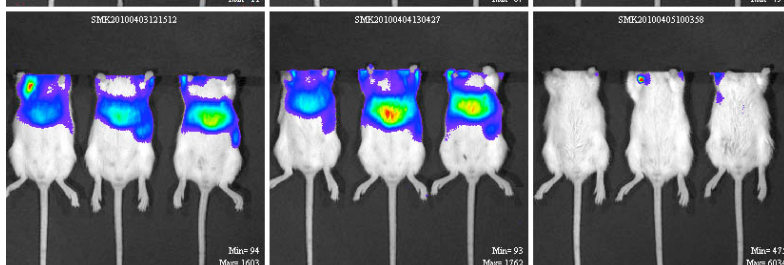

Supplement: Additional file 1 — Images of mice infected with IHD-J-Luc and either treated or not with anti-QVPA cocktail. Mice described in the legend to Figure 4 were subjected to whole-body imaging daily for 10 days or during survival. Representative sequential images of mice 1, 2, and 3 from control group (PBS only, panels A and C) and of mice 1, 2, and 3 from the group that received QVPA (panels B and D) are shown. Images of heads (A, B) were acquired from the same mice as images of the torso (C, D) but using shorter exposure to avoid saturation of the camera due to high signal from the nasal cavity. Images from control and from treated mice were collected on day 1-6 (A, C) and on day 1-9 (B, D), respectively. [file 1743-422X-8-441-S1.PDF]
